# Assessing depression among the Japanese population: psychometric properties of the Japanese version of Quick Inventory of Depressive Symptomatology-self-reported version

**DOI:** 10.1186/s40359-026-04974-9

**Published:** 2026-06-16

**Authors:** Masami Kashimura, Mirai So, Yoichi Sekizawa

**Affiliations:** 1 Tokiwa University, 1-430-1, Miwa, Mito, Ibaraki, 310-8585 Japan; 2 Ichikawa General Hospital, International University of Health and Welfare, 5-11-13, Sugano, Ichikawa, Chiba, Japan; 3 The Research Institute of Economy, Trade, and Industry, 1-3-1, Kasumigaseki, Chiyoda, Tokyo, Japan

**Keywords:** Depression, Quick Inventory of Depressive Symptomatology, Factor structure, Measurement invariance, Reliability, Validity

## Abstract

**Background:**

The Quick Inventory of Depressive Symptomatology (QIDS) is a useful, validated measure of depressive symptoms that covers the DSM-IV diagnostic criteria for major depressive disorder. The Japanese translation of the QIDS (QIDS-J) has been published, but its psychometric properties have not been validated in large samples.

**Methods:**

This study aimed to examine the psychometric properties of the QIDS-J using secondary analysis data from a Japanese clinical study of internet-based cognitive-behavioral therapy that includes 1,187 participants (698 males, 489 females; mean age: 43.46 ± 9.85). First, a cross-sectional survey examined descriptive statistics, factor structure, measurement/structural invariance, internal consistency, construct validity, and age and gender differences in measurement scores. Subsequently, a short-term longitudinal and cross-sectional survey was conducted to evaluate test–retest reliability.

**Results:**

Explanatory and confirmatory factor analyses revealed a one-factor structure that demonstrated good internal consistency and test–retest reliability, as well as partially verified concurrent and construct validity.

**Conclusions:**

These findings indicate that the QIDS-J has robust psychometric properties and can assess depressive symptoms across nine DSM-IV criterion domains.

**Trial registration:**

University Hospital Medical Information Network Clinical Trials Registry (UMINCTR) UMIN000019228 (Registration Date October 4, 2015) https://www.center6.umin.ac.jp/cgiopenbin/ctr_e/ctr_view.cgi?recptno=R000022220.

## Background

Major depressive disorder (MDD) is common, with almost one in five people experiencing an episode at some point in their lifetime [1]. Moreover, subthreshold depression, defined as having a clinically relevant level of depressive symptoms without meeting the diagnostic criteria for a major depressive disorder [2], has an estimated prevalence of 11.02% [3]. Thus, a great number of individuals experience and suffer from depressive symptoms. Early detection of depression is important not only in the field of psychiatry but also for improving treatment outcomes in patients with physical illnesses (e.g., rheumatoid arthritis), and the use of appropriate assessment tools is essential [4]. Furthermore, since the early detection and treatment of depression can ameliorate its negative effects [5], it is crucial to detect individuals suffering from depressive symptoms and intervene as soon as possible. Tools that assess the presence and severity of depressive symptoms are therefore important not only in primary care settings but also in the general population.

Self-rated questionnaires are a common instrument for assessing depressive symptoms. Compared to clinician-rated measures, such as the Hamilton Rating Scale for Depression (HRSD) [6], self-rated questionnaires are inexpensive in terms of professional time needed for incorporation into the clinical setting since they do not require any special training for use. Such tools are also free of clinician bias and the risk of clinician overestimation of patient improvement, and these correlate highly with clinician ratings [7]. Accordingly, many self-rated measures have been developed to assess depressive symptoms, with many translations available worldwide. Some representative examples include the Beck Depression Inventory-II (BDI-Ⅱ) [8], Self-rating Depression Scale [9], Center for Epidemiologic Studies Depression Scale [10], Hospital Anxiety and Depression Scale (HADS) [11], Patient Health Questionnaire‐9 (PHQ-9) [12], Depression Anxiety Stress Scales-21 [13], and Quick Inventory of Depressive Symptomatology (QIDS) [14]. In particular, the QIDS was uniquely developed based on all DSM-IV [15] diagnostic criteria for MDD, and it evaluates both the presence and severity of depressive symptoms [14]. As with other self-rated questionnaires, the QIDS has more than 30 language versions developed worldwide. Since the criteria for major depressive episode are almost identical in the DSM-Ⅳ and DSM-5 [16], the QIDS therefore remains a relevant and useful assessment tool.

The QIDS has two versions: a clinician rating scale (QIDS-C) and a self-report scale (QIDS-SR). The QIDS-SR was designed to assess depression severity and evaluate 16 types of depressive symptoms that meet the DSM-IV diagnostic criteria for MDD: falling asleep, sleep during the night, waking up too early, sleeping too much, feeling sad, decreased or increased appetite, decreased or increased weight, concentration/decision making, view of myself, thoughts of death or suicide, general interest, energy level, feeling slowed down, and feeling restless. This measure assesses symptoms within the past seven days (independent of whether they have been long-term, chronic, or recent). In the QIDS scoring system, the responses to these 16 items are classified under the 9 DSM-IV symptom criterion domains: (1) sad mood, (2) concentration, (3) self-criticism, (4) suicidal ideation, (5) interest, (6) energy/fatigue, (7) sleep disturbance (i.e., initial, middle, and late insomnia or hypersomnia), (8) decrease/increase in appetite/weight, and (9) psychomotor agitation/retardation [14]. Each item was rated on a 4-point scale (0–3), with the QIDS total score ranging from 0 to 27. Scoring criteria have been established, wherein a total calculated QIDS score of 6–10 is classified as mild (79% sensitivity, 81% specificity, 74% positive predictive value, and 85% negative predictive value), 11–15 as moderate, 16–20 as severe, and 21–27 as very severe [14].

Rush et al. [14] examined the psychometric properties of QIDS with 681 adult outpatients (age 18–75) diagnosed with MDD or dysthymic disorder. Rush et al. [14, 17] reported the unidimensionality of QIDS, revealing the good internal consistency of the QIDS-SR (α = 0.86) and its strong correlations with other measures for depression (*rs* ≥ 0.81). Furthermore, the QIDS-SR was found to have item-total correlations of *r* ≥ 0.60, except for sleep disturbance (*r* = 0.52) and appetite/weight change (*r* = 0.49). Similar results were found in another study as well [17]. Thereafter, Reilly et al. [18] conducted a meta-analysis of previous QIDS studies and reviewed the literature. Various subsequent studies have shown that both QIDS-C and QIDS-SR exhibit a stable one-factor structure and good internal consistency (αs = 0.69–0.89). However, regarding item-total correlations, several studies showed sleep disturbance and appetite/weight change in the QIDS-SR to be below *r* = 0.30 [18]. In addition, correlations between the QIDS-SR and other depression measures ranged from *rs* = 0.17–0.86. The most frequently examined correlation was between QIDS-SR and the 17-item HRSD [6], yielding moderate-to-high correlations (*rs* = 0.68–0.96).

The Japanese version of the QIDS-SR (QIDS-J) has been developed [19], and its reliability and validity have been reported [19]. The QIDS-J has been examined for 29 outpatients (72.4% female) diagnosed with major depression or dysthymic disorder based on DSM-Ⅳ (age 18–65), and its reliability and validity have been verified [19]. Additionally, the QIDS-J has been used in various clinical studies [20] and surveys [21]. However, the factor structure has not been examined, and its reliability and validity within a large sample have not been clearly established. This rigorous evaluation is particularly critical because mental health outcomes are not merely internal psychological states but rather arise from dynamic interactions between an individual’s adaptive capacities and broader interpersonal, organizational, and cultural contexts [22]. Validating the QIDS-J in a large Japanese sample provides a theoretical foundation for culturally sensitive research and mental health promotion.

### The present study

This study aimed to examine the psychometric properties of the QIDS-J. First, the factor structure was examined through both explanatory factor analysis (EFA) and confirmatory factor analysis (CFA). Specifically, we tested whether the QIDS-J exhibited a one-factor structure, as previous studies have reported. Since measurement invariance for QIDS-SR remains unknown, it was examined in this study as well. Second, reliability was measured using internal consistency and the test–retest method. In particular, the test–retest reliability of QIDS-SR is highly significant because no previous reports have discussed this [18]. Third, concurrent and construct validity were assessed using the PHQ-9 [12], Generalized Anxiety Disorder-7 (GAD-7) [23], the General Trust Scale (GTS-5) [24], the Sheehan Disability Scale (SDS) [25], and subjective satisfaction with life and home.

The review of Reilly et al. [18] found that the correlations between the QIDS-SR and PHQ-9 were *rs* = 0.73–0.75, and thus the expected correlation of the QIDS-J is estimated at *r* = 0.70. Considering that depression and anxiety are closely related [26], Cameron et al. [27] reported that the QIDS-SR and HADS-Anxiety component had a correlation of *r* = 0.70. Therefore, the QIDS-J is similarly hypothesized to have a correlation of *r* = 0.70. Subsequently, the correlation between the QIDS-SR-J and GTS-5 was examined. Several studies have reported associations between low levels of trust and MDD [28]. The correlation between GTS-5 and depression, as measured by the BDI-Ⅱ, was *r* = − 0.22 [29]. Therefore, the correlation between the QIDS-J and GTS-5 was estimated at *r* = − 0.20. Additionally, we also examined the correlation between the QIDS-J and SDS, a measure of social disability. Since the SDS (social disability) correlated with PHQ-9 scores at *r* = 0.41 [30], the QIDS-J and SDS were hypothesized to correlate at a similar level. Lastly, the correlation between the QIDS-J and subjective satisfaction with life and home was examined. Since life satisfaction has been previously correlated with depression, as measured by the BDI-Ⅱ, at *r* = 0.60 [31], the QIDS-J and subjective satisfaction were hypothesized to correlate at approximately the same level.

## Methods

This study was conducted in accordance with the Declaration of Helsinki. This study was approved by the Hiramatsu Memorial Hospital Ethics Committee (approval number 20150807) and registered in the UMIN Clinical Trials registry system (UMIN000019228; Registration Date October 4, 2015). All participants completed a consent form, provided explicit consent online, and were informed that participation in this study was voluntary and that they could withdraw at any time.

### Procedure and participants

This study evaluated the psychometric properties of the QIDS-SR-J using a data set from a previous 12-week randomized controlled trial comparing internet-based cognitive behavioral therapy with artificial intelligence, internet-based cognitive behavioral therapy, and the waiting-list among the Japanese general population [32]. The inclusion criteria for participation in this data set were: (1) age 20–60 years at entry (i.e., working-age population), (2) available Internet access, (3) at least five points on the PHQ-9 at screening, and (4) capacity to understand and agree to the Japanese-language instructions. The exclusion criteria were as follows: (1) presence of medical conditions precluding participation as determined by a physician, (2) concurrent participation in other forms of cognitive behavioral therapy, (3) diagnosis of schizophrenia, (4) diagnosis of dementia, (5) severe suicidal ideation, and (6) substance dependence in the past 12 months (excluding smoking).

Potential participants from the above clinical trial by So et al. [32] were recruited to a survey panel at Nikkei Research Inc. The consent explanatory document of the study was presented online, and participants freely provided explicit consent online. Data on each measure was collected at three time points: at baseline in September 2015 (Time 1 data), postintervention in November 2015 (Time 2 data), and at follow-up in February 2016 (Time 3 data). As an honorarium for the study participants, those who completed the questionnaires at Times 2 and 3 (but not at Time 1) received a gift voucher worth 500 yen (maximum 1,000 yen). This was communicated to participants during the process of written explanation of the consent form. Participants received brief instructions on study participation and links to the web-based questionnaire. The study explanation and informed consent details were presented on the first page of the questionnaire form. Participants were assured that their responses would remain confidential. Those who agreed to participate were allowed to answer the questionnaire at any time of their choice. Participants who indicated willingness to participate in follow-up surveys (Times 2 and 3) were asked to provide their e-mail addresses, with clarification that the information would not be used to identify individuals. Participants were informed that they could withdraw at any time without penalty. Only the authors had access to the study data.

Out of 1,249 participants, 1,187 adults who agreed to participate in this study (698 male, 489 female; mean ± standard deviation [SD] age, 43.46 ± 9.85 years; range, 20–60), were asked about each measurement at baseline and regarding their demographic data. All procedures for recruitment and obtaining consent for participation were conducted during September 2015). At baseline, individuals in this dataset who did not meet the above inclusion criteria were included. All participants were asked to answer all questions, and thus there were no missing values in this data set.

To examine the retest reliability of the QIDS-J, we extracted a sample of 394 participants (subsample C: 234 male, 160 female; mean ± SD age, 43.57 ± 10.12 years; range, 20–60) who were assigned to the waiting-list group at Time 1 in So et al. [32]. The other groups were not suitable for examining the time stability of the QIDS-J scores due to the implementation of psychological interventions between Time 1 and Time 2. So et al. [32] reported that the total sample was assigned to three groups (two intervention groups and one waiting group), with no significant differences in demographic data, depression, or anxiety scores across groups. Thus, 300 participants (182 male, 118 female; mean ± SD age, 43.42 ± 9.95 years; range, 20–60) were followed through Time 2 and Time 3.

### Measures

The following sociodemographic characteristics were recorded: age, gender, education, occupation, marital status, and history of general practice or psychiatric visits.

The QIDS-J is a self-reported measure that assess depressive symptoms through 16 items rated on a 3-point Likert scale: sleep disturbance (items 1–4), sad mood (item 5), change in appetite or weight (items 6–9), concentration/decision making (item 10), self-view (item 11), suicidal ideation (item 12), general interest (item 13), energy/fatigue (item14), and psychomotor agitation/retardation (items 15–16). The total scores for items 1–4, 6–9, and 15–16 were used as the scores for sleep disturbance, appetite/weight, and psychomotor agitation/retardation, respectively. Those four scores, alongside the remaining items, are summed to form the QIDS-J total score; this ranges from 0 to 27, with higher scores indicating more depressive symptoms.

The PHQ-9 [12] is a 9-item self-report measure for depressive symptoms, rated by a 4-point Likert scale (0 = not at all; 3 = nearly every day), with higher scores indicating greater depression. The PHQ-9 has previously demonstrated good reliability and validity [12, 33, 34], and our sample exhibited good internal consistency (α = 0.86).

The GAD-7 [23] is a 7-item self-reported measure for anxiety that occurred over the last 2 weeks, rated by a 4-point Likert scale (0 = not at all; 3 = nearly every day), with higher scores indicating greater anxiety. The Japanese version was used [35]. The GAD-7 has previously demonstrated good reliability and validity [23, 36], and our sample showed good internal consistency (α = 0.88).

The GTS-5 is a 5-item self-reported measure that assesses participants’ beliefs about trust in others [24] across a 7-point Likert scale (1 = strongly disagree; 7 = strongly agree), with higher scores indicating stronger trust in others. It has previously been validated [24], and our sample showed good internal consistency (α = 0.93).

The SDS [25] is a 3-item self-report measure for disability across 3 domains (work and schoolwork, social and leisure activities, and family life and home responsibilities) that uses an 11-point Likert scale (0 = no disability; 10 extreme disability), with higher scores indicating greater disability. We used the Japanese version [37]. It has previously demonstrated good reliability and validity [38, 39], and our sample showed good internal consistency (α = 0.92).

In addition, participants were asked regarding satisfaction in daily life (“How satisfied are you now with your life in general?”) and satisfaction with one’s own household (“How satisfied are you now with your family?”). Participants were asked to rate their satisfaction on an 11-point scale (0 = very unsatisfactory, 10 = very satisfactory), with higher scores indicating greater satisfaction.

### Data analysis

The mean ± SD was calculated for each variable score using Time 1 data. Statistical significance was set at *p* < 0.05 for the following analyses. To examine potential age and gender differences in the QIDS-J, we conducted an analysis of covariance (ANCOVA) using QIDS-J scores as the dependent variable, gender as the independent variable, and age as a covariate.

To assess the factor structure of the QIDS-J, the Time 1 sample (*n* = 1,187) was randomly divided into 2 subsamples. EFA was conducted with the first sample (subsample A, *n* = 593; 368 male, 225 female; mean ± SD age, 43.64 ± 10.09 years; range, 20–60), then CFA was conducted with the second sample (subsample B, *n* = 594; 330 male, 264 female; mean ± SD age, 43.28 ± 9.60 years; range, 20–60). In EFA, the number of factors was estimated using the visual scree test [40], parallel analysis, and minimum average partials (MAP). The maximum likelihood method was used for factor extraction. We included items without cross-load over multiple factors and with factor loadings > 0.35 on a primary factor, considering that meaningful factor loadings range from 0.30 to 0.40 [41].

In the CFA, the scale parameter was estimated via maximum likelihood estimation. Model fit was determined based on a previous study that conducted a confirmatory factor analysis of the QIDS [42] using the Comparative Fit Index (CFI), Tucker–Lewis index (TLI), and root mean square error of approximation (RMSEA). CFI or TLI values of ≥ 0.95 and RMSEA values of ≤ 0.05 suggest a good fit, whereas CFI or TLI values of 0.90–0.94 and RMSEA values of 0.07–0.10 suggest an acceptable fit. Although a power analysis of sample size was not conducted for EFA and CFA, the sample size was deemed sufficient based on the COSMIN checklist criteria (number of items × 7 and at least 100 participants) [43]. After conducting each factor analysis, the inter-item correlations of the QIDS-J and item-total correlations were calculated for the total sample.

Multi-group CFA was performed to test the measurement invariance of the QIDS-SR-J across gender (male and female) and age group (younger and older) in subsample B (*n* = 594). Since the overall age range was 20–60 years in this study, the younger age group was defined as 20–39 years old (*n* = 217; 105 male, 112 female; mean ± SD, 32.66 ± 4.77 years), while the older age group was set at 40–60 years old (*n* = 377; 225 male, 152 female; mean ± SD, 49.40 ± 5.46 years). The age cut-off of 40 years was selected to reflect the institutional and sociocultural framework of the Japanese health system. In Japan, age 40 marks a significant transition point for health management in the workforce, as it is the age when individuals become eligible for national ‘Specific Health Checkups’ (Tokutei Kenshin) and ‘Specific Health Guidance’ programs aimed at preventing lifestyle-related diseases [44]. Thus, age 40 serves as a recognized threshold for health awareness and preventive care in the Japanese general population [45]. The following four models were progressively tested with more invariance constraints regarding whether these were equal across groups: configural (model 1; structure of the factor), metric (model 2; factor loadings for each item), scalar (model 3; intercepts of each item) and strict invariances (model 4; error variances for each item) [46]. The model was constrained to maintain equality across groups for factor loadings, item intercepts, and factor variances. Additionally, to assess structural invariance, we examined factor variance invariance (model 5; factor variances) and mean invariance (a mean-difference analysis of latent variables). Since a one-factor structure was expected for QIDS-J, factor covariance invariance was not examined. Measurement invariance was determined based on the results of the chi-square test, wherein a significant difference indicated that measurement invariance was not met. However, since the chi-square difference test is sensitive to sample size [47], the change in CFI (*Δ*CFI) was also used to indicate measurement invariance, wherein *Δ*CFI ≤ 0.01 indicated measurement invariance [48].

To assess reliability, Cronbach’s alpha was calculated for the Time 1 data, determined as either acceptable reliability (α = 0.70–0.80) or good reliability (α = 0.80) [49, 50]. The test–retest method was applied and intraclass correlations (ICC) [2, 3] were calculated to evaluate time stability using data across the 3 time points for the 394 participants (subsample C) assigned to the waiting group (no intervention) in the data set. This is because intervention groups, other than the waiting group, are more likely to show changes in their QIDS-J scores. The approximate time interval was 2 months between Time 1 and Time 2, whereas this was 3 months between Time 2 and Time 3. For the ICC criteria, values of 0.40–0.59, 0.60–0.74, and > 0.75 were considered fair, good, and excellent, respectively [49].

To examine construct validity, Pearson correlation coefficients were calculated using Time 1 data, assessing associations between QIDS-J scores and scores on other measures. The PHQ-9, GAD-7, GTS-5, SDS, and subjective satisfaction were used to examine the validity of the QIDS-J. Lastly, hierarchical multiple regression analysis was performed to assess whether the QIDS-J provided additional predictive value beyond that of the PHQ-9. SDS was used the dependent variable. The demographic data was initially entered as independent variables (Step 1), followed by the PHQ-9 (Step 2), and finally the QIDS-J (Step 3). All procedures were conducted using a forced-entry regression.

HAD version 18.0 [51] was used for EFA, parallel analysis, and calculating MAP. CFA was conducted using IBM SPSS AMOS version 26. Other analyses were conducted using IBM SPSS version 30 (IBM, Armonk, NY, USA).

## Results

Table 1 presents the demographics of the study cohort, while Table 2 presents descriptive statistics for the variables in this study. The mean scores for the nine domains of the QIDS-J did not differ markedly from each of the mean scores reported by Rush et al. [17]. The results of ANCOVA indicated that age significantly affected QIDS-J scores (*F* [1, 1184] = 4.61, *p* = 0.032, partial *n*^*2*^ = 0.004), whereas gender had no significant effect (*F* [1, 1184] = 1.80, *p* = 0.179, partial *n*^*2*^ = 0.002). Younger age was correlated with depressive symptoms (*B* = − 0.03, standard error [*SE*] = 0.01, *t* = 2.15, *p* = 0.032, partial *n*^*2*^ = 0.004).

**Table 1:** Demographic data

Characteristic	Value
Mean age(*SD*)	43.46 (9.85)
Range	20–60
Gender (%)	
Male	698 (58.8)
Female	489 (41.2)
Education (%)	
Junior high school	7 (0.6)
High school	239 (20.1)
Junior college/Technical college/Vocational school	216 (18.2)
University/Graduate School	725 (61.1)
Occupation (%)	
Employed	972 (81.9)
Unemployed and seeking employment	79 (6.7)
Unemployed	136 (11.5)
Marriage (%)	
Married	665 (56.0)
Divorced	76 (6.4)
Bereavement	7 (0.6)
Unmarried	439 (37.0)
History of medical visits for mental disorders (%)	
Currently under treatment	129 (10.9)
Received treatment in the past but not currently being treated	184 (15.5)
Never received treatment	874 (73.6)
History of medical visits for physical illness (%)	
Not under treatment	918 (77.3)
Undergoing treatment on an outpatient basis	258 (21.7)
Hospitalized and under treatment	11 (0.9)
Number of outpatient visits in last 30 days (%)	
None	657 (55.3)
More than 1 day but less than 3 days	445 (37.5)
More than 4 days but less than 9 days	57 (4.9)
More than 10 days	28 (2.4)

*SD* standard deviation

**Table 2:** Descriptive statistics and Cronbach’s alpha coefficients of all variables

	Total sample (*N* = 1,187)			Male (*n* = 698)		Female (*n* = 489)	
	M	SD	Range	M	SD	M	SD
QIDS-J							
Total score ^a^ (*α* = 0.82)	8.73	4.85	0–27	8.54	4.99	8.99	4.62
Item 1	1.01	0.95	0–3	0.96	0.95	1.08	0.95
Item 2	1.23	0.97	0–3	1.26	0.98	1.20	0.96
Item 3	0.81	1.00	0–3	0.90	1.01	0.69	0.97
Item 4	0.30	0.63	0–3	0.26	0.61	0.35	0.66
Sleep disturbance	1.69	0.94	0–3	1.68	0.96	1.70	0.91
Item 5 (sad mood)	0.94	0.77	0–3	0.88	0.77	1.03	0.76
Item 6	0.31	0.57	0–3	0.34	0.61	0.27	0.50
Item 7	0.60	0.97	0–3	0.45	0.82	0.81	1.11
Item 8	0.29	0.68	0–3	0.34	0.73	0.22	0.60
Item 9	0.52	0.92	0–3	0.47	0.88	0.61	0.96
Change in appetite or weight	1.15	1.05	0–3	1.04	1.01	1.31	1.09
Item 10 (concentration/decision making)	0.87	0.72	0–3	0.87	0.75	0.86	0.68
Item 11 (self-view)	0.89	0.96	0–3	0.88	0.96	0.92	0.98
Item 12 (suicidal ideation)	0.62	0.78	0–3	0.60	0.78	0.65	0.78
Item 13 (general interest)	0.92	0.89	0–3	0.91	0.88	0.92	0.90
Item 14 (energy/fatigue)	0.92	0.64	0–3	0.91	0.64	0.93	0.64
Item 15	0.58	0.68	0–3	0.62	0.70	0.52	0.66
Item 16	0.45	0.67	0–3	0.50	0.69	0.38	0.65
Psychomotor agitation/retardation	0.73	0.75	0–3	0.77	0.74	0.67	0.74
PHQ-9 total (*α* = 0.86)	8.74	5.19	0–27	8.40	4.10	11.00	6.08
GAD-7 total (*α* = 0.88)	6.03	4.60	0–21	6.00	4.00	7.67	6.03
GTS-5 total (*α* = 0.93)	18.65	6.32	5–35	21.40	4.39	21.33	1.53

*QIDS-J* Japanese version of Quick Inventory of Depressive Symptomatology-Self-Report

^a^ The QIDS-J total score was calculated by summing its nine-domain scores

EFA was performed on subsample A (*n* = 593) to determine the number of factors in the QIDS-J. The visual scree test revealed a one-factor solution, with eigenvalues of 3.87 and 0.96 for factors 1 and 2, respectively. The parallel analysis and MAP indicated a one-factor solution. The results of the EFA (maximum likelihood method, no rotation) revealed that each QIDS-J item had factor loadings of 0.35 or higher (Table 3). The one-factor solution explained 43.03% of the variance of QIDS-J scores. Subsequently, CFA was conducted on subsample B (*n* = 594) to verify the one-factor structure of the QIDS-J. In line with a previous meta-analysis of the QIDS [18], the one-factor model showed mostly acceptable model fit (CFI = 0.92, TLI = 0.90, RMSEA = 0.08). Table 4 shows the correlation coefficients between items of the QIDS-J (*rs* = 0.21–0.50). In addition, the QIDS-J item-total correlations were calculated, revealing the following: sleep disturbance (0.36), sad mood (0.53), appetite/weight (0.41), concentration/decision making (0.61), self-view (0.61), suicidal ideation (0.54), general interest (0.59), energy/fatigue (0.56), and psychomotor agitation/retardation (0.58).

**Table 3:** Explanatory factor analysis and confirmatory factor analysis of the QIDS-J

	Subsample A (*n* = 593)	Subsample B (*n* = 594)
	Factor loadings	Factor loadings ^a, b^
QIDS-J		
1. Sleep disturbance	0.37	0.39
2. Sad mood	0.63	0.57
3. Appetite/weight changes	0.46	0.43
4. Concentration/decision making	0.69	0.68
5. Self-view	0.71	0.66
6. Suicidal ideation	0.67	0.53
7. General interest	0.67	0.66
8. Energy/fatigue	0.61	0.65
9. Psychomotor agitation/retardation	0.67	0.64

*QIDS-J* Japanese version of Quick Inventory of Depressive Symptomatology-Self-Report

^a^ Standardized factor loadings

^b^ All factor loadings are statistically significant (*p* < 0.001)

**Table 4:** Pearson correlations between each domain of the QIDS-J

	1	2	3	4	5	6	7	8
1. Sleep disturbance	-							
2. Sad mood	0.21	-						
3. Appetite/weight changes	0.26	0.21	-					
4. Concentration/decision making	0.26	0.39	0.32	-				
5. Self-criticism	0.24	0.46	0.31	0.46	-			
6. Suicidal ideation	0.22	0.50	0.21	0.36	0.47	-		
7. General interest	0.26	0.39	0.29	0.46	0.44	0.39	-	
8. Energy/fatigue	0.27	0.32	0.30	0.45	0.38	0.32	0.48	-
9. Psychomotor agitation/retardation	0.23	0.37	0.29	0.50	0.44	0.37	0.41	0.44

Sample size, *n* = 1,187

*QIDS-J* Japanese version of Quick Inventory of Depressive Symptomatology-Self-Report

All significance levels were *p* < 0.001

Multi-group CFA was used to determine the measurement invariance of the QIDS-J across gender and age group (Table 5). The configural, metric, scalar, and strict invariances models were progressively tested and evaluated using the *Δ*CFI. For the comparisons between age groups, although the metric invariance yielded satisfactory values on fit indices, the *Δ*CFI between model 2 and model 3 was above the threshold of ≤ 0.01, indicating that full scalar invariance was not supported. In addition, all models showed good fit across generation groups, and none showed significant chi-square differences. Thus, full measurement invariance of the QIDS-J across age groups was achieved. To test for structural invariance, factor variances were constrained to be equal between age groups. The *Δ*CFI between model 4 and model 5 was below the threshold of ≤ 0.01, indicating an acceptable level of structural invariance. Lastly, the latent mean differences between different group means on a construct were estimated. After fixing the factor means of one group to zero (the means of the other group were freely estimated), the differences in factor means between groups were determined, using the fixed group (the younger group) as the reference group. No significant difference was found between both age groups (estimate of the mean = − 0.02, *SE* = 0.03, *z* statistic [estimate of mean/*SE*] = − 0.47, *p* = 0.639).

**Table 5:** Measurement invariance across gender/generations of the QIDS-J

Model	χ^2^ (df)^a^	CFI	TLI	RMSEA (90% CI)	χ^2^ (df) difference test	*p* value	ΔCFI
Gender							
1. Configural	146.74 (54)	0.93	0.91	0.05 (0.04–0.06)			
2. Metric	152.42 (62)	0.93	0.92	0.05 (0.04–0.06)	5.68 (8)	0.683	−0.001
3. Scalar	189.84 (71)	0.91	0.91	0.05 (0.04–0.06)	37.42 (9), *p*	< 0.001	0.020
4. Strict	247.01 (80)	0.88	0.89	0.06 (0.05–0.07)	57.17 (9), *p*	< 0.001	0.035
5. Factor variance/covariance^b^	247.27 (81)	0.88	0.89	0.06 (0.05–0.07)	0.26 (1)	0.611	0.000
Generations							
1. Configural	161.61 (54)	0.92	0.90	0.06 (0.05–0.07)			
2. Metric	170.68 (62)	0.92	0.91	0.05 (0.05–0.06)	9.08 (8)	0.336	0.000
3. Scalar	185.36 (71)	0.92	0.92	0.05 (0.04–0.06)	14.68 (9)	0.100	0.005
4. Strict	196.39 (80)	0.92	0.92	0.05 (0.04–0.06)	11.03 (9)	0.274	0.001
5. Factor variance/covariance^b^	196.48 (81)	0.92	0.93	0.05 (0.04–0.06)	0.09 (1)	0.761	−0.001

*QIDS-J *Japanese version of Quick Inventory of Depressive Symptomatology-Self-Report,* CFI* comparative fit index, *TLI* Tucker-Lewis index, *RMSEA* root mean square error of approximation, *CI* confidence interval

^a^ For all models, *χ*^*2*^ *p* < 0.001

^b^ Only the factor variance was constrained because the QIDS-J has a one-factor structure

The reliability and validity of the QIDS-J was evaluated. It demonstrated good internal consistency reliability (Table 2), as well as excellent ICC [2, 3] across all three time points (ICC = 0.84, *p* < 0.001, 95% confidence interval [CI], 0.81–0.87). As hypothesized, the QIDS-J score significantly correlated with the PHQ-9, GAD-7, GTS-5, SDS, and subjective satisfaction (Table 6). The QIDS-J scores demonstrated a significant positive correlation with the PHQ-9, GAD-7, and SDS, as well as significant negative correlations with the GTS-5 and with two types of subjective satisfaction.

**Table 6:** Pearson correlations between QIDS-J scores and other variable scores

	QIDS-J	1	2	3	4	5
1. PHQ-9	0.76					
	[0.73, 0.78]					
2. GAD-7	0.72	0.78				
	[0.69, 0.74]	[0.75, 0.80]				
3. GTS-5	−0.27	−0.26	−0.26			
	[− 0.32, − 0.21]	[− 0.31, − 0.20]	[− 0.32, − 0.21]			
4. SDS	0.57	0.54	0.57	−0.18		
	[0.53, 0.61]	[0.50, 0.58]	[0.53, 0.61]	[− 0.23, − 0.12]		
5. Life satisfaction	−0.41	−0.42	−0.36	0.23	−0.43	
	[− 0.46, − 0.36]	[− 0.47, − 0.37]	[− 0.41, − 0.31]	[0.17, 0.28]	[− 0.47, − 0.38]	
6. Satisfaction with family life	−0.35	−0.36	−0.31	0.16	−0.40	0.65
	[− 0.39, − 0.29]	[− 0.41, − 0.31]	[− 0.36, − 0.26]	[0.11, 0.22]	[− 0.45, − 0.35]	[0.62, 0.68]

Sample size, *n* = 1,187

*QIDS-J* Japanese version of Quick Inventory of Depressive Symptomatology-Self-Report, *PHQ-9* Patient Health Questionnaire-9, *GAD-7* Generalized Anxiety Disorder-7, *GTS-5* General Trust Scale (5 items), *SDS* Sheehan Disability Scale

All significance levels were *p* < 0.001; [ ] indicate the 95% confidence intervals

Hierarchical multiple regression analysis revealed the predictive potential of the QIDS-J for the SDS (Table 7). After all demographic data and the PHQ-9 were forced in as predictors (Steps 1 and 2), the QIDS-J was forced in (Step 3). This resulted in a significant increase in *R*^*2*^ values (*ΔR*^*2*^ = 0.06, *p* < 0.001), indicating that the QIDS-J scores could significantly explain the SDS scores (*β* = 0.37, *p* < 0.001).

**Table 7:** Hierarchical multiple regression analysis

	β		*R* ^2^	ΔR^2^	
Step 1			0.07	0.07	^***^
Age	−0.08	^*^			
Gender	0.01				
Education	−0.05				
Occupation	−0.01				
Marriage	−0.04				
History of medical visits for mental disorders	−0.23	^***^			
History of medical visits for physical illness	−0.01				
Number of outpatient visits in last 30 days	0.08	^*^			
Step 2			0.31	0.24	^***^
PHQ-9	0.51	^***^			
Step 3			0.37	0.06	^***^
QIDS-J	0.37	^***^			

Sample size, *n* = 1,187

*QIDS-J* Japanese version of Quick Inventory of Depressive Symptomatology-Self-Report, *PHQ-9* Patient Health Questionnaire-9, *SDS* Sheehan Disability Scale, *β* Standardized beta coefficient

^***^p < 0.001; ^**^p < 0.01; ^*^p < 0.05

## Discussion

This study aimed to examine the QIDS-J and evaluate its psychometric properties. The QIDS-J was confirmed to have a one-factor structure, consistent with previous studies [14, 17, 18]. Both the EFA and CFA showed relatively low factor loadings for sleep disturbance and appetite/weight changes on the QIDS-J, consistent with the results of Rush et al. [17], but all item factor loadings were above the criterion value. The mean scores for most items on the QIDS-J were close to zero, and nearly identical results were reported by Rush et al. [14]. Although significant positive correlations were found across the nine domains of QIDS-J, sleep disturbance and appetite/weight changes demonstrated relatively weaker correlations with the other domains, consistent with previous studies as well [17, 52]. Collectively, the results of this study largely replicated those of the original QIDS-SR. In addition to this psychometric consistency, the specific nature of physical symptoms—characterized by low factor loadings and weak correlations—may be understood from the perspective of an individual’s emotion regulation. According to previous research, a lack of cognitive reappraisal or dependence on emotion suppression may increase physiological stress and influence the manifestation and reporting of physical symptoms [53]. Therefore, clinical interventions targeting these emotion regulation skills may also influence the reporting and management of depressive symptoms, potentially providing a broader clinical perspective on the assessment of depression.

The measurement/structural invariances of the QIDS-J were examined across gender and age groups. The age groups were found to have the same structure (i.e., same number of factors), item factor loadings, item intercepts, item error variances, and factor variance for the QIDS-J. In addition, no significant differences across age groups were found in the latent means of the QIDS-J. These findings suggest that the measured values provided by the QIDS-J are not influenced by age. However, the measurement invariance across gender was only established at the level of metric invariance with the same structure and factor loadings for each item. The results of this study on QIDS-J measurement invariance could be crucial, since they indicate that QIDS-J scores can be compared across samples. This study represents the first attempt to examine the measurement/structural invariance of QIDS-SR. Differences in invariance across other groups (e.g., country, culture, ethnic groups) will require further investigation. Conceptualizations and experiences of mental health are strongly dependent on sociocultural contexts, and there are limitations to applying universal (one-size-fits-all) models directly [22]. The perspective of the “Cultural Integration Model,” in which individual, interpersonal, organizational, and cultural levels dynamically interact, is essential for promoting mental health [22]. In this study, the rigorous examination of the psychometric properties of the QIDS-J within the specific cultural context of Japan provides a theoretical foundation for developing clinical interventions and policies tailored to that society.

In terms of reliability, Cronbach’s alpha coefficients for the QIDS-J scores were good. These results are largely consistent with those of Rush et al. [14] and Fujisawa et al. [19] and are approximately consistent with the meta-analysis of the QIDS [18]. In addition, the ICC values of QIDS-J scores were excellent. Although the test–retest reliability of the QIDS-SR has not yet been reported [18], QIDS-J scores were relatively stable over time in this study. This study is significant because it further strengthens the findings on the psychometric properties of the QIDS-SR.

Regarding validity, QIDS-J scores were significantly correlated with the hypothesized relevant variables, demonstrating that the construct validity of the QIDS-J was supported. The QIDS-J scores demonstrated a strong positive correlation with the PHQ-9 scores, consistent with the review of Reilly et al. [18]. This finding further supports the concurrent validity of the QIDS-J. Further, we found strong positive correlations between the QIDS-J and the GAD-7. Depression and anxiety are known to be closely related [26], and Cameron et al. [27] reported correlations between the QIDS-SR and HADS (a measurement of anxiety symptoms), consistent with our findings. Moderate positive correlations were also observed between the QIDS-J and SDS, consistent with the report of Kroenke et al. [30]. The SDS is a validated measure of functional outcome in MDD [54] that is widely used in clinical trials to assess changes in functional impairment in patients with MDD. Patients with MDD are impaired not only in the ability to perform activities of daily living but also in diverse domains such as interpersonal skills and work capacity/productivity [55], and thus the moderate correlations between the QIDS-J and SDS in this study are considered a reasonable result. However, negative correlations were observed between the QIDS-J, GTS-5, and two types of subjective satisfaction with life or family. Since low levels of trust are associated with MDD [28], our results were consistent with those of a previous study [29] and support the hypothesized association. Furthermore, despite the weak to moderate correlations between the QIDS-J and satisfaction with life or family, these were somewhat lower than those shown in a previous study [31]. In that study, life satisfaction was measured using a 4-item scale (score range: 4–20), whereas this study used a single-item satisfaction scale (score range: 0–10). This difference in correlations could be attributed to the different methods used to measure satisfaction. Considering the above, we therefore conclude that the series of results presented in this study is valid.

On hierarchical multiple regression analysis, the QIDS-J scores significantly predicted functional impairments measured via SDS, even after controlling for demographic data and PHQ-9 scores. The incremental predictive value of the QIDS-J relative to the SDS underscores the importance of validating scales within specific clinical contexts to ensure high sensitivity to treatment outcomes [56]. Generic tools may overlook the multidimensional concepts essential for assessing functional recovery. Furthermore, functional impairment is often mediated by psychological resources such as self-efficacy and adaptive coping strategies [57]. The QIDS-J’s ability to assess diverse symptom domains may more effectively capture the underlying psychological mechanisms that lead to functional disability, thereby offering a more precise and context-sensitive evaluation of patient outcomes. The QIDS-J may predict or explain more functional impairment than the PHQ-9, although its predictive validity remains to be demonstrated. Nevertheless, further research is needed to clarify the superior aspects of the QIDS-J compared to other depression scales.

Finally, given that the data for this study were obtained through an internet-based randomized controlled trial involving AI-based cognitive behavioral therapy, the nature of AI-assisted assessment warrants consideration. While traditional clinical evaluations remain the gold standard, recent evidence suggests that AI-driven systems can provide highly reliable and objective assessments of complex human states and skills. For example, an AI-assisted assessment of interpersonal communication skills has been shown to demonstrate nearly perfect agreement with human raters (ICC = 0.97–0.99) and to provide consistent, unbiased feedback [58]. In the present study, the use of AI-assisted platforms for data collection may enhance the scalability and objectivity of psychological assessments, thereby supporting the psychometric robustness of the QIDS-J even in digital health environments.

This study has some limitations. First, although the study cohort included a relatively large sample of general adults (aged 20–60 years) in Japan, clinical groups were not distinguished. However, Fujisawa et al. [18] examined the cutoff value for remission of depression using the QIDS-J, and several studies have also reported treatment responsiveness alongside other measures of depression [17]. In the present study, treatment responsiveness and clinician-rated QIDS were not addressed due to the lack of data based on an accurate diagnosis of depression. Further studies are needed to evaluate the clinical usefulness of the QIDS-J using large-scale data. Another limitation is that the present study was a secondary analysis of data from an existing study [30], and the sample did not include data from the HRSD [5], which is considered the standard measure for assessing depression. Thus, this study could not demonstrate an association between the QIDS-J and HRSD, which is used as a validity measure in most QIDS studies. Lastly, although the measurement/structural invariance of the QIDS-J was examined across different age groups (20–39 years and 40–60 years), these groupings were arbitrary, thereby limiting its accuracy.

## Conclusion

This study examined the psychometric properties of the QIDS-J in detail, specifically including factor structure, measurement/construct invariance, reliability, and validity. Although the QIDS-J has been used in various studies in Japan without adequate psychometric evaluation, our results indicate that it could be used to assess depressive symptoms. Despite revisions in the diagnostic criteria for mental disorders, the DSM-IV criteria for MDD remain largely unchanged in the current DSM-5, and the value of the QIDS thus remains undiminished. In summary, the QIDS is a highly convenient self-administered questionnaire that can be useful for assessing both the presence and severity of depressive symptoms, with fewer items than conventional questionnaires for assessing depressive symptoms.

## Data Availability

The data used in this study belong to the Research Institute of Economy, Trade and Industry. Due to the commitment to the study participants, the data collected for this study cannot be shared publicly.

## Abbreviations

ANCOVA: An analysis of covariance
BDI-Ⅱ: Beck Depression Inventory-II
CFA: Confirmatory factor analysis
CFI: Comparative Fit Index
CI: Confidence interval
EFA: Explanatory factor analysis
GAD-7: Generalized Anxiety Disorder-7
GTS-5: General Trust Scale
HADS: Hospital Anxiety and Depression Scale
HRSD: Hamilton Rating Scale for Depression
ICC: Intraclass correlations
MAP: Minimum average partials
MDD: Major depressive disorder
QIDS: Quick Inventory of Depressive Symptomatology
QIDS-C: Quick Inventory of Depressive Symptomatology: a clinician rating scale
QIDS-J: Japanese version of the Quick Inventory of Depressive Symptomatology: a self–report scale
QIDS-SR: Quick Inventory of Depressive Symptomatology: a self–report scale
RMSEA: Root mean square error of approximation
PHQ-9: Patient Health Questionnaire-9
SD: Standard deviation
SE: Standard error
SDS: Sheehan Disability Scale
TLI: Tucker–Lewis index
